# ﻿Novel *Helicotubeufia* and *Tubeufia* (Tubeufiaceae, Tubeufiales) species from terrestrial habitats in Hainan Province, China

**DOI:** 10.3897/mycokeys.126.174148

**Published:** 2025-12-04

**Authors:** Ting-Hong Tan, Fan Gao, Song Bai, Chun-Fang Wu, Ning-Ning Zhao, Na Qiu, Min Zhou, Jian Ma

**Affiliations:** 1 School of Agriculture and Forestry Engineering and Planning, Tongren University, Tongren, Guizhou 554300, China; 2 Guizhou Provincial Key Laboratory for Biodiversity Conservation and Utilization in the Fanjing Mountain Region, Tongren University, Tongren, Guizhou 554300, China; 3 Guizhou Industry Polytechnic College, Guiyang, Guizhou 550008, China; 4 School of Food and Pharmaceutical Engineering, Guizhou Institute of Technology, Guiyang, Guizhou 550003, China

**Keywords:** Asexual morph, phylogeny, saprobic fungi, taxonomy, two new species

## Abstract

Helicosporous hyphomycetes are a group of fungi, characterised by coiled or spiral conidia and are known for their potential to produce bioactive secondary metabolites. During a survey of helicosporous hyphomycetes, four isolates were obtained from decaying wood in Hainan Province, southern China. Based on phylogenetic analyses of a combined dataset (ITS, LSU, *tef*1-α and *rpb*2) and morphological characteristics, two novel species, *Helicotubeufia qixianlingensis* and *Tubeufia diaoluoshanensis*, are introduced. Comprehensive descriptions, illustrations and phylogenetic analyses supporting the taxonomic placement of these new taxa are provided. Notably, *H. qixianlingensis* represents the first record of *Helicotubeufia* from a terrestrial habitat, thereby expanding the known diversity of tropical terrestrial fungi.

## ﻿Introduction

Liu et al. (2018) introduced the new genus *Helicotubeufia* Y.Z. Lu & J.K. Liu to accommodate three species, *H. guangxiensis*, designated as the type species, alongside *H. hydei* and *H. jonesii*, based on phylogenetic analyses of combined ITS, LSU and *tef*1-α sequences data and morphological features. These three species were obtained from submerged decaying wood in China and Thailand (Liu et al. 2018). Recently, Ma et al. (2024) identified an additional species within the *Helicotubeufia*, named *H. laxisporum*, which was also isolated from submerged decaying wood in Hainan Province, China. Thus, the genus *Helicotubeufia* now encapsulates four recognised species (Lu et al. 2018b; Ma et al. 2024). The asexual morph of *Helicotubeufia* is characterised by hyaline, macronematous conidiophores, holoblastic, polyblastic, sympodial, hyaline conidiogenous cells and helicoid, hyaline conidia (Liu et al. 2018). The sexual morph is characterised by superficial, seated on a subiculum, scattered, subglobose to ellipsoidal-ovate ascomata, 8-spored, bitunicate asci and fusiform, hyaline ascospores (Liu et al. 2018).

*Tubeufia* was established by Penzig and Saccardo (1897) with *T. javanica* as the type species, based on morphological characteristics. The genus currently comprises 88 species, including 29 from freshwater habitats, 44 from terrestrial habitats and 15 occurring in both freshwater and terrestrial habitats (Ma et al. 2023, 2024; 2025; Lu et al. 2025). *Tubeufia* species are widely distributed, having been reported from Austria, Bermuda, Brazil, Canada, China, Colombia, Cuba, India, New Zealand, Panama, Peru, South Africa, Sri Lanka, Tanzania, Thailand, Trinidad, Uganda, Venezuela and the USA (Morgan 1892; Talbot 1956; Moore 1957; Rao and Rao 1964; Deighton and Pirozynski 1966; Munk 1966; Ellis 1971; Panwar et al. 1973; Barr 1979, 1980; Barr and Rogerson 1983; Goos 1985, 1990; Matsushima 1987, 1993; Rossman 1987; Holubová-Jechová 1988; Karandikar and Patwardhan 1992; Lu et al. 2000; Chang 2001, Ho et al. 2002; Tsui and Berbee 2006; Tsui 2007; Zhao et al. 2007; Pande 2008; Boonmee et al. 2011, 2014, 2021; Hyde et al. 2016; Singh and Singh 2016; Chaiwan et al. 2017; Dai et al. 2017; Doilom et al. 2017; Lu et al. 2017, 2018a, 2018b, 2022, 2023; Luo et al. 2017; Kuo and Goh 2018a, 2018b, 2021; Tibpromma et al. 2018; Li et al. 2022; Tian et al. 2022; Ma et al. 2023, 2024; 2025; Lu et al. 2025). The conidial morphology in *Tubeufia* is highly diverse, encompassing a variety of forms such as dictyosporous, muriform, dorsiventrally curved, coiled, ovate, ellipsoid to ovoid, spherical to obclavate and subreniform forms, sometimes accompanied by one or more small, globose secondary conidia (Tsui and Berbee 2006; Tsui 2007; Zhao et al. 2007; Lu et al. 2018b; Ma et al. 2023, 2024).

In this study, four hyphomycete isolates were obtained from terrestrial habitats in Hainan Province, China, specifically from Diaoluoshan National Nature Reserve and Qixianling Hot Spring National Forest Park. Based on morphological observations, detailed illustrations, descriptive notes and multi-gene phylogenetic analyses, we propose two novel species: *Helicotubeufia qixianlingensis* and *Tubeufia diaoluoshanensis*. Comparative analyses with closely-related taxa further substantiate their taxonomic classification.

## ﻿Materials and methods

### ﻿Sample collection, specimen examination and isolation

Decaying wood was collected from Hainan Province, south-western China. Samples were taken to the laboratory in plastic bags with the collection details, including localities and dates. The microscopic features were examined and photographed using a stereomicroscope (SMZ-168, Nikon, Japan) and an ECLIPSE Ni compound microscope (Nikon, Tokyo, Japan) with a Canon 90D digital camera (Canon, China). Measurements were made by Tarosoft (R) Image Frame Work software. Photo plates were assembled using Adobe Photoshop CC 2019 (Adobe Systems, USA).

Single spore isolation was performed following the methods described by Senanayake et al. (2020) and the germinated conidia were aseptically transferred to fresh PDA plates. Morphological characters of fungal mycelia, including colour, shape and size, were documented. Dried fungal specimens were deposited in the Herbarium of Kunming Institute of Botany, Chinese Academy of Sciences (Herb. HKAS) in Kunming, China and the Herbarium of Guizhou Academy of Agriculture Sciences (Herb. GZAAS), Guiyang, China. Pure cultures were deposited in the Guizhou Culture Collection (GZCC), Guiyang, China. MycoBank numbers of newly-obtained species were registered in the MycoBank database (https://www.mycobank.org/).

### ﻿DNA extraction, PCR amplification and sequencing

Fresh fungal mycelia were scraped from colonies grown on PDA plates and transferred to a 1.5 ml microcentrifuge tube using a sterilised lancet for genomic DNA extraction. Genomic DNA was extracted using the Biospin Fungus Genomic DNA Extraction Kit (BioFlux, China). ITS5/ITS4, LR0R/LR5, EF1-983F/EF1-2218R and fRPB2-5F/fRPB2-7cR were employed to amplify the internal transcribed spacer (ITS; White et al. (1990)), large ribosomal subunit (LSU; Vilgalys and Hester (1990)), translation elongation factor 1-alpha (*tef*1-α; Rehner and Buckley (2005)) and RNA polymerase II second largest subunit (*rpb2*; Liu et al. (1999)) sequence fragments, respectively. DNA preparation was conducted in a 25 μl mixture, which included 1 μl DNA, 1 μl of the forward and reverse primers each and 22 μl of 1.1× T3 Super PCR Mix (including 8.5 μl distilled-deionised water; Qingke Biotech, Chongqing, China). The conditions for the polymerase chain reaction (PCR) correspond to those reported by Ma et al. (2023). The PCR products were purified and sequenced with the same primers at Beijing Tsingke Biotechnology Co., Ltd.

### ﻿Genealogical concordance phylogenetic species recognition (GCPSR) analysis

The pairwise homoplasy index (PHI) test of *Helicotubeufia* species was carried out in SplitsTree4 (Bruen et al. 2006). It indicates that there is no statistically significant evidence for recombination for the selected taxa when the P-value is above 0.05. Both the LogDet transformation and splits decomposition options were used to reveal the relationship amongst closely-related species.

### ﻿Phylogenetic analyses

The newly-obtained sequences were checked and assembled using BioEdit v.7.0.5.3 (Hall 1999) and SeqMan v.7.0.0 (DNASTAR, Madison, WI, USA; Swindell and Plasterer (1997)), respectively. The sequences incorporated in this study were downloaded from GenBank (Table 1; https://www.ncbi.nlm.nih.gov/). Multiple sequences were aligned using MAFFT v.7.473 (https://mafft.cbrc.jp/alignment/server/; Katoh et al. (2019)). The dataset was trimmed using trimAl v.1.2rev59 software (Capella-Gutiérrez et al. 2009). A combined sequence dataset was created using SequenceMatrix-Windows-1.7.8 software (Vaidya et al. 2011).

**Table 1.** Taxa used in this study and their GenBank accession numbers of DNA sequences.

Taxon	Strain	GenBank Accessions			
		ITS	LSU	*tef*1-α	*rpb*2
*Acanthohelicospora aurea*	GZCC 16-0060	KY321323	KY321326	KY792600	MF589911
*Acanthohelicospora guianensis*	UAMH 1699	AY916479	AY856891	N/A	N/A
*Helicotubeufia guangxiensis*	MFLUCC 17-0040^T^	MH290018	MH290023	MH290028	MH290033
*Helicotubeufia hydei*	MFLUCC 17-1980^T^	MH290021	MH290026	MH290031	MH290036
*Helicotubeufia jonesii*	MFLUCC 17-0043^T^	MH290020	MH290025	MH290030	MH290035
*Helicotubeufia laxisporum*	CGMCC 3.25545^T^	PP626589	PP639445	PP596346	PP596473
*Helicotubeufia qixianlingensis*	GZCC 25-0644	PX575636	PX575659	PX512842	PX512833
*Helicotubeufia qixianlingensis*	GZCC 25-0645^T^	PX575635	PX575658	PX512841	PX512832
*Tubeufia abundata*	MFLUCC 17-2024	MH558769	MH558894	MH550961	MH551095
*Tubeufia acropleurogena*	CGMCC 3.25582^T^	PP626645	PP639501	PP596394	PP596513
*Tubeufia africana*	BCRC FU30906	LC371247	LC424099	N/A	LC494221
*Tubeufia aquatica*	MFLUCC 17-1794	MH558770	MH558895	MH550962	MH551096
*Tubeufia bambusicola*	MFLUCC 17-1803^T^	MH558771	MH558896	MH550963	MH551097
*Tubeufia baomeilingensis*	CGMCC 3.25580^T^	PP626648	PP639504	PP596397	PP596515
*Tubeufia brevis*	MFLUCC 17-1799^T^	MH558772	MH558897	MH550964	MH551098
*Tubeufia brunnea*	MFLUCC 17-2022^T^	MH558773	MH558898	MH550965	MH551099
*Tubeufia chiangmaiensis*	MFLUCC 11-0514^T^	KF301530	KF301538	KF301557	N/A
*Tubeufia chlamydospora*	MFLUCC 16-0223^T^	MH558775	MH558900	MH550967	MH551101
*Tubeufia cocois*	MFLUCC 22-0001^T^	OM102541	OL985957	OM355486	OM355491
*Tubeufia cylindrothecia*	MFLUCC 16-1283	KY320518	KY320535	KY320552	MH551143
*Tubeufia denticulata*	CGMCC 3.25583^T^	PP626653	PP639509	PP596402	PP596520
*Tubeufia diaoluoshanensis*	GZCC 22-2142	PX575637	PX575660	PX512843	PX512834
*Tubeufia diaoluoshanensis*	GZCC 25-0643^T^	PX575638	PX575661	PX512844	PX512835
*Tubeufia dictyospora*	MFLUCC 17-1805^T^	MH558778	MH558903	MH550970	MH551104
*Tubeufia dongfangensis*	GZCC 22-2125^T^	PQ098479	PQ098516	N/A	N/A
*Tubeufia eccentrica*	MFLUCC 17-1524^T^	MH558782	MH558907	MH550974	MH551108
*Tubeufia entadae*	MFLU 18-2102^T^	MK347727	MK347943	N/A	N/A
*Tubeufia fangchengensis*	MFLUCC 17-0047^T^	MH558783	MH558908	MH550975	MH551109
*Tubeufia filiformis*	MFLUCC 16-1128^T^	N/A	KY092407	KY117028	MF535284
*Tubeufia formosiformis*	BCRC FU30757^T^	LC193730	LC201751	N/A	LC494220
*Tubeufia formosiformis*	BCRC FU30851^T^	LC371250	LC424102	N/A	LC494218
*Tubeufia freycinetiae*	MFLUCC 16-0252^T^	MH275089	MH260323	MH412786	N/A
*Tubeufia geniculata*	BCRC FU30849^T^	LC335817	N/A	N/A	N/A
*Tubeufia guangxiensis*	MFLUCC 17-0045^T^	MG012025	MG012018	MG012004	MG012011
*Tubeufia guttulata*	GZCC 23-0404^T^	OR030841	OR030834	OR046678	OR046684
*Tubeufia hainanensis*	GZCC 22-2015^T^	OR030842	OR030835	OR046679	OR046685
*Tubeufia hechiensis*	MFLUCC 17-0052^T^	MH558785	MH558910	MH550978	MH551112
*Tubeufia hyalospora*	MFLUCC 15-1250^T^	MH558786	MH558911	MH550979	N/A
*Tubeufia inaequalis*	MFLUCC 17-0053^T^	MH558789	MH558914	MH550982	MH551115
*Tubeufia javanica*	MFLUCC 12-0545^T^	KJ880034	KJ880036	KJ880037	N/A
*Tubeufia jianfenglingensis*	GZCC 23-0021^T^	PQ098481	PQ098518	N/A	N/A
*Tubeufia krabiensis*	MFLUCC 16-0228^T^	MH558792	MH558917	MH550985	MH551118
*Tubeufia latispora*	MFLUCC 16-0027^T^	KY092417	KY092412	KY117033	MH551119
*Tubeufia laxispora*	MFLUCC 16-0232^T^	KY092413	KY092408	KY117029	MF535287
*Tubeufia lilliputea*	NBRC 32664	AY916483	AY856899	N/A	N/A
*Tubeufia liyui*	GZCC 22-2030^T^	OP888466	OP888465	OP856589	OP856588
*Tubeufia longihelicospora*	MFLUCC 16-0753^T^	MZ538531	MZ538565	MZ567106	N/A
*Tubeufia longiseta*	MFLUCC 15-0188^T^	KU940133	N/A	N/A	N/A
*Tubeufia machaerinae*	MFLUCC 17-0055	MH558795	MH558920	MH550988	MH551122
*Tubeufia mackenziei*	MFLUCC 16-0222^T^	KY092415	KY092410	KY117031	MF535288
*Tubeufia muriformis*	GZCC 22-2039^T^	OR030843	OR030836	OR046680	OR046686
*Tubeufia nigroseptum*	CGMCC 3.20430^T^	MZ092716	MZ853187	OM022002	OM022001
*Tubeufia pandanicola*	MFLUCC 16-0321^T^	MH275091	MH260325	N/A	N/A
*Tubeufia parvispora*	MFLUCC 16-0324^T^	MH275090	MH260324	MH412787	MH412761
*Tubeufia roseohelicospora*	MFLUCC 15-1247^T^	KX454177	KX454178	N/A	MH551144
*Tubeufia rubra*	GZCC 16-0081^T^	MH558801	MH558926	MH550994	MH551128
*Tubeufia sahyadriensis*	NFCCI 4252/RAJ 99.1^T^	MH033849	MH033850	MH033851	N/A
*Tubeufia sessilis*	MFLUCC 16-0021^T^	MH558803	N/A	MH550996	MH551130
*Tubeufia subrenispora*	CGMCC 3.25560^T^	PP781938	PP781939	PP785815	PP785813
*Tubeufia sympodihylospora*	MFLUCC 17-0044^T^	MH558806	MH558930	MH550999	MH551133
*Tubeufia sympodilaxispora*	MFLUCC 17-0048^T^	MH558808	MH558932	MH551001	MH551135
*Tubeufia taiwanensis*	BCRC FU30844^T^	LC316605	N/A	N/A	N/A
*Tubeufia tectonae*	MFLUCC 12-0392^T^	KU144923	KU764706	KU872763	N/A
*Tubeufia tratensis*	MFLUCC 17-1993^T^	MH558811	MH558935	MH551004	MH551138
*Tubeufia tropica*	GZCC 23-0219^T^	PP626675	PP639531	N/A	PP596536
*Tubeufia xylophila*	MFLUCC 17-1520	MH558813	MH558937	MH551006	MH551140
*Tubeufia xylophila*	GZCC 16-0038	MH558812	MH558936	MH551005	MH551139
*Tubeufia yanuodaensis*	GZCC 23-0488^T^	PQ098484	PQ098521	PV768323	PV768332
*Tubeufia yinggelingensis*	GZCC 23-0525^T^	PQ098483	PQ098520	PV768321	PV768330
Tubeufiaceae sp.	BCC 3512	AY916484	AY856905	N/A	N/A

Note: “^T^” denotes ex-type strain. Newly generated sequences are indicated in bold black. “N/A” means no data available in GenBank.

The Maximum Likelihood (ML) analysis was carried out using the RAxML-HPC v.8 on XSEDE (8.2.12) tool using a GTRGAMMA approximation with rapid bootstrap analysis followed by 1000 bootstrap replicates (Stamatakis 2014). The substitution model was automatically tested by the server. Bayesian Inference (BI) analysis was performed by using MrBayes on XSEDE (3.2.7a) via CIPRES (Stamatakis 2014). The aligned FASTA file was converted to a Nexus format file using AliView (Daniel et al. 2010). The best-fit evolutionary model for the individual dataset was determined using MrModelTest v. 2.3. 10 (Nylander et al. 2008). The GTR+G+I substitution model was selected for ITS, LSU, *tef*1-α and *rpb*2. The posterior probabilities (BYPP) were determined, based on Bayesian Markov Chain Monte Carlo (BMCMC) sampling (Huelsenbeck and Ronquist 2001). Two simultaneous Markov chains were run for 10,000,000 generations and trees were sampled every 1,000^th^ generation. The burn-in phase was set at 25% and the remaining trees were used for calculating posterior probabilities (BYPP).

Phylogenetic trees were visualised using FigTree v.1.4.4 and edited with Adobe Illustrator CC 2019 (v.23.1.0; Adobe Systems, USA).

## ﻿Phylogenetic results

The phylogenetic placements of the newly-isolated taxa were determined by multi-locus phylogenetic analysis. A total of 70 strains, including our newly-isolated strains and two outgroups, were analysed. The concatenated sequence matrix consisted of 3,365 characters (ITS = 565 bp, LSU = 843 bp, *tef*1-α = 912 bp and *rpb*2 = 1,045 bp). Base frequencies and rates were A = 0.248200, C = 0.249485, G = 0.256312 and T = 0.246003; substitution rates were AC = 1.017042, AG = 5.690331, AT = 2.161337, CG = 0.779562, CT = 8.363479 and GT = 1.000000. The distribution shape parameter α equalled 0.179497.

**Figure 1.**
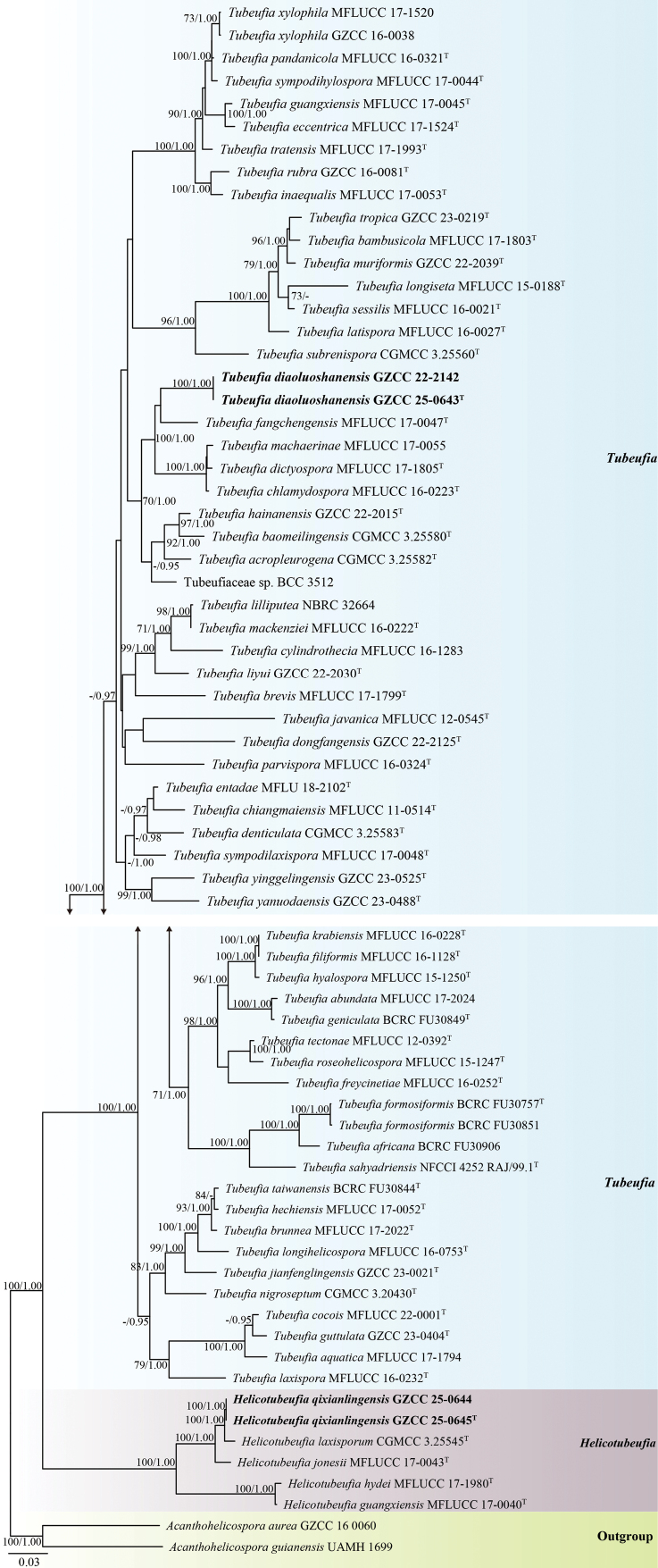
Phylogenetic tree generated from the Maximum Likelihood (ML) analysis, based on a combined dataset of ITS, LSU, *tef*1-α and *rpb*2 sequence data. Bootstrap support values from ML analyses ≥ 55% and Bayesian posterior probabilities (BYPP) ≥ 0.95 are indicated at the nodes as ML/BYPP, respectively. The Maximum Likelihood (ML) and Bayesian Inference (BYPP) analyses yielded similar tree topologies. Hyphen (“-”) indicates a value lower than 55% for ML and a posterior probability lower than 0.95 for Bayesian. The tree is rooted with *Acanthohelicospora aurea*GZCC 16-0060 and *A. guianensis* UAMH 1699. The newly-obtained strains are indicated in black bold. Ex-type strains are denoted with “^T^”.

Based on the phylogenetic analysis (Fig. 1), our collections belong to *Helicotubeufia* and *Tubeufia* within Tubeufiaceae (Tubeufiales, Dothideomycetes). Two isolates (GZCC 25-0644 and GZCC 25-0645) formed a sister clade with *Helicotubeufia laxisporum* (CGMCC 3.25545), supported by 100% ML and 1.00 BYPP. Additionally, GZCC 22-2142 and GZCC 25-0643 form a sister lineage to *Tubeufia fangchengensis* (MFLUCC 17-0047) with 55% ML support.

### ﻿Taxonomy

#### ﻿ Helicotubeufia qixianlingensis

Taxon classificationFungiTubeufialesTubeufiaceae

T.H. Tan & J. Ma sp. nov.

A8166274-C04F-552B-BAF1-9EFE3FAB3884

904431

Fig. 2

##### Etymology.

“*qixianlingensis*” refers the place “Qixianling Hot Spring National Forest Park” from where the fungus was collected.

**Figure 2.**
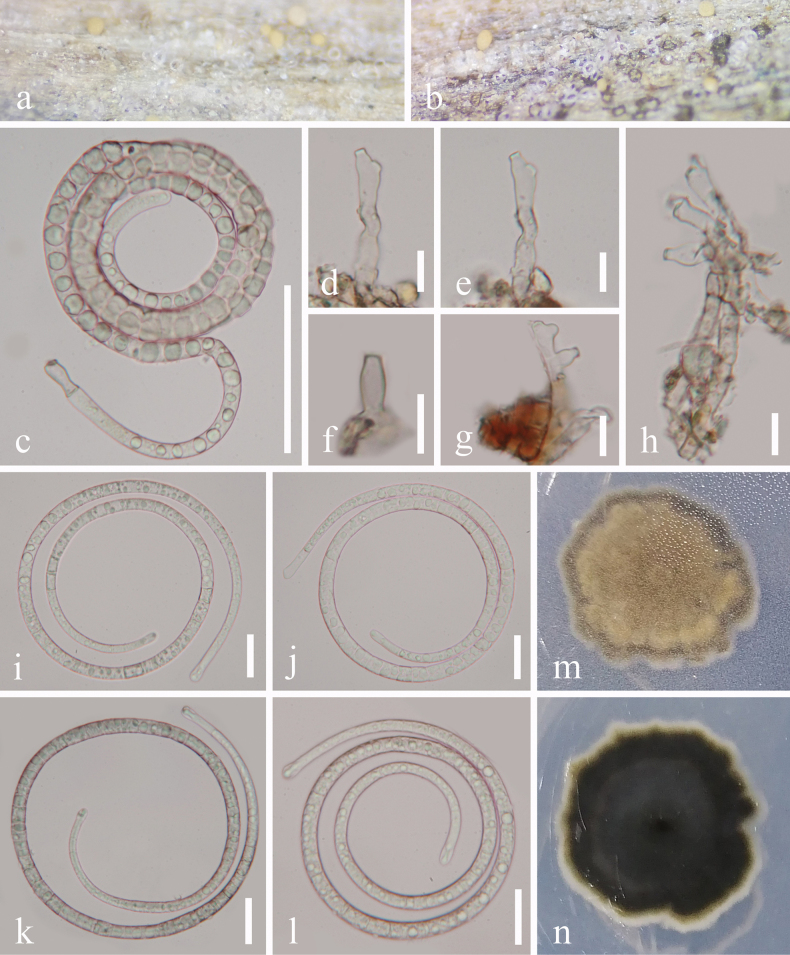
*Helicotubeufia qixianlingensis* (GZAAS 25-0674, holotype). a, b. Colonies on the host surface; c. Conidiophores and conidia; d–h. Conidiophores; i–l. Conidia; m, n. Colonies on PDA, m from above, n from below. Scale bars: 50 μm (c); 20 μm (i–l); 10 μm (d–h).

##### Holotype.

GZAAS 25-0674.

##### Description.

***Saprobic*** on decaying wood in a terrestrial habitat. ***Sexual morph*** Undetermined. ***Asexual morph*** Hyphomycetous, helicosporous. ***Colonies*** on natural substrate superficial, effuse, gregarious, with masses of crowded, glistening conidia, white to pale brown. ***Mycelium*** partly immersed, partly superficial, composed of hyaline to pale brown, branched, septate, arising from creeping hyaphae, guttulate, smooth hyphae. ***Conidiophores*** 13–38 × 4.5–5.5 μm (*x̄* = 28 × 5 μm, n = 25), macronematous, mononematous, solitary or cespitose, erect, irregular cylindrical, short, flexuous, branched or unbranched, septate, pale brown, smooth-walled, thick-walled. ***Conidiogenous cells*** holoblastic, monoblastic or polyblastic, integrated, terminal, cylindrical, truncate at apex after conidial secession, pale brown, smooth-walled. ***Conidia*** solitary, acrogenous, helicoid, tapering towards the rounded ends, 77–101 μm diameter and conidial filament 6–7 μm wide (*x̄* = 90 × 6.5 μm, n = 20), 483–520 μm long (*x̄* = 499 μm, n = 20), loosely coiled 2–2^1^/_2_ times, becoming loosely coiled in water, indistinctly multi-septate, subhyaline to pale brown, smooth-walled.

##### Culture characteristics.

Conidia germinated on PDA and produced germ tubes within 9 h. Colonies on PDA reached 26 mm in diameter after 38 days of incubation at 25 °C with an irregular shape, flat surface and undulate margin, pale brown to brown; the reverse was brown to black.

##### Material examined.

China • Hainan Province, Baoting Li and Miao Autonomous County, Qixianling Hot Spring National Forest Park, on decaying wood in a terrestrial habitat, 2 November 2024, Jian Ma, Q26 (GZAAS 25-0674, holotype), ex-type living culture GZCC 25-0645; • *Ibid*., Q28 (GZAAS 25-0673, paratype), living culture GZCC 25-0644.

##### Notes.

**Figure 3.**
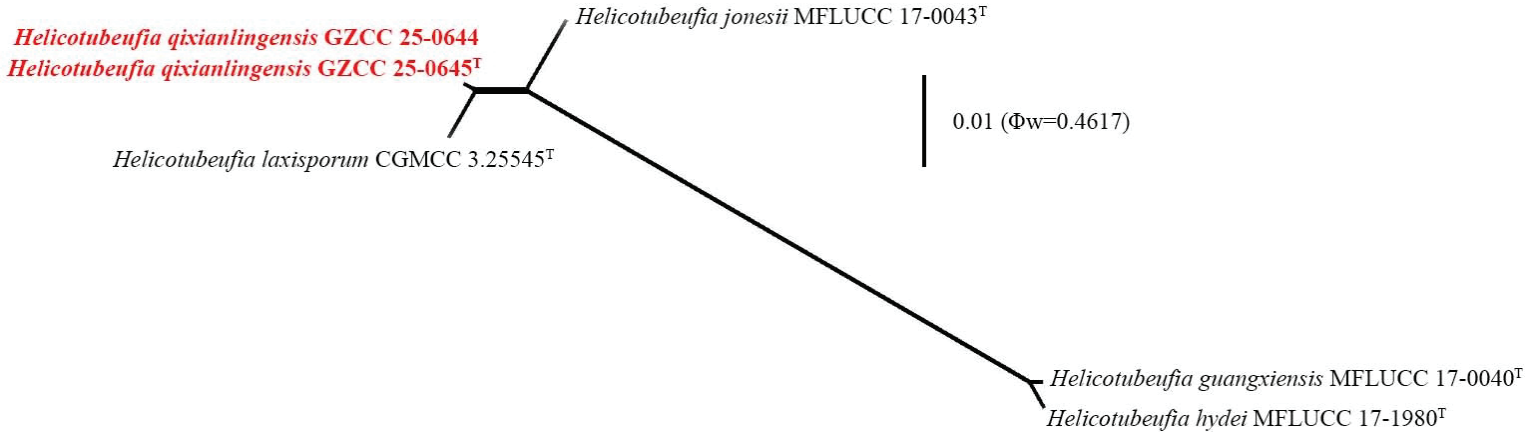
Results of the PHI test of *Helicotubeufia qixianlingensis* (GZCC 25-0644 and GZCC 25-0645) with closely-related species (combined LSU-ITS-*tef*1-α-*rpb*2) using both LogDet transformation and splits decomposition. PHI test results (Φw) < 0.05 indicate significant recombination within the dataset. New species are indicated in red bold and type strains are marked with “^T^”.

In the phylogenetic analysis (Fig. 1), *Helicotubeufia qixianlingensis* (GZCC 25-0644 and GZCC 25-0645) formed a sister lineage to *H. laxisporum* (CGMCC 3.25545) with 100% ML and 1.00 BYPP statistical support. Sequence comparisons revealed that our isolates (GZCC 25-0645, ex-type) differ from *H. laxisporum* (CGMCC 3.25545) by 17/517 bp in ITS (3.3%, including eight gaps), 2/861 bp in LSU (0.2%, no gaps), 3/914 bp in *tef*1-α (0.3%, no gaps) and 0/1,097 bp in *rpb*2 (0%, no gaps). Morphologically, *H. qixianlingensis* (GZAAS 25-0674) differs from *H. laxisporum* (HKAS 128907) by its shorter conidiophores (13–38 × 4.5–5.5 μm vs. up to 59 × 5–8.5 μm) and a greater number of conidial times (2–2^1^/_2_ vs. 1^1^/_3_–2). In addition, the PHI test results (Fig. 3) revealed no significant recombination relationships between *Helicotubeufia qixianlingensis* (GZCC 25-0644 and GZCC 25-0645) and its phylogenetically related taxa. Therefore, *Helicotubeufia qixianlingensis* is introduced here as a new species, based on morphological and phylogenetic analyses, with supporting evidence from the PHI test.

#### ﻿ Tubeufia diaoluoshanensis

Taxon classificationFungiTubeufialesTubeufiaceae

T.H. Tan & J. Ma sp. nov.

32EF01A9-D142-5F99-93F4-3399EBD9F747

904432

Fig. 4

##### Etymology.

“*diaoluoshanensis*” refers the place “Diaoluoshan National Nature Reserve” from where the fungus was collected.

**Figure 4.**
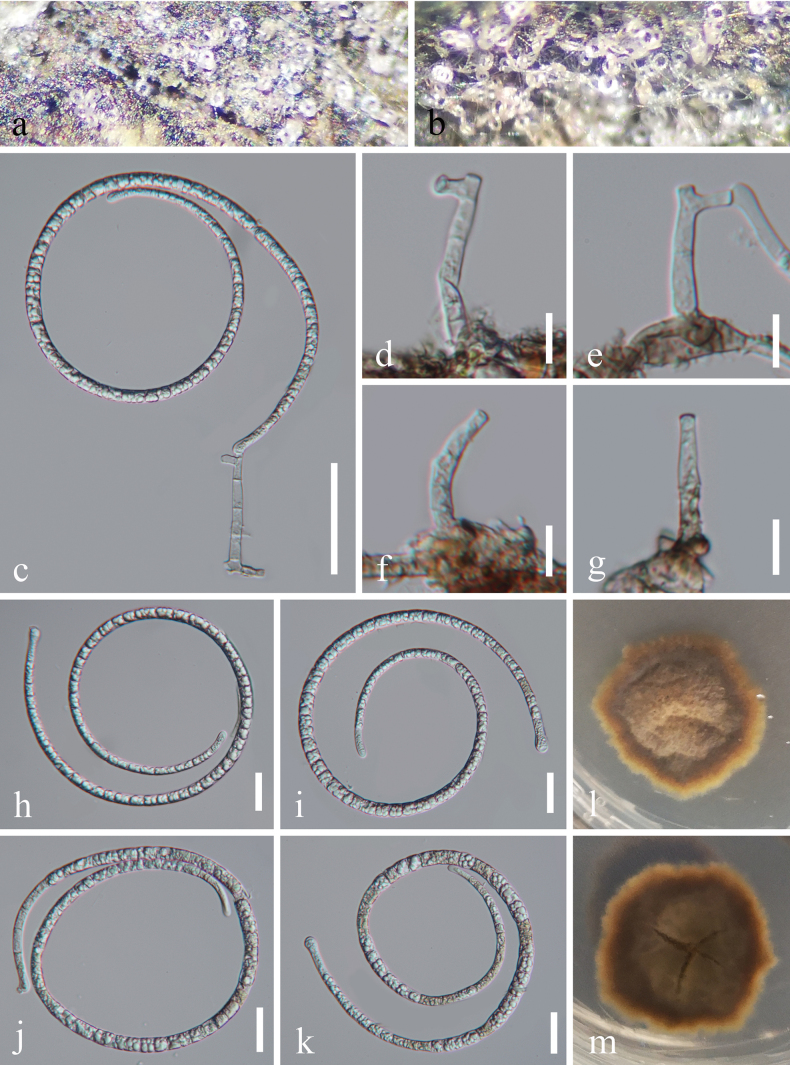
*Tubeufia diaoluoshanensis* (HKAS 128911, holotype). a, b. Colonies on the host surface; c Conidiophores, conidiogenous cells and conidia; d–g. Conidiophores and conidiogenous cells; h–k. Conidia; l, m. Colonies on PDA, l from above, m from below. Scale bars: 50 μm (c); 20 μm (h–k); 10 μm (d–g).

##### Holotype.

HKAS 128911.

##### Description.

***Saprobic*** on decaying wood in a terrestrial habitat. ***Sexual morph*** Undetermined. ***Asexual morph*** Hyphomycetous, helicosporous. ***Colonies*** on natural substrate superficial, effuse, solitary, gregarious, with mass of crowded, glistening conidia, white to pale brown. ***Mycelium*** partly immersed, partly superficial, composed of hyaline to pale brown, branched, septate, smooth hyphae. ***Conidiophores*** 24–57 × 4–6 μm (*x̄* = 36.5 × 4.5 μm, n = 20), macronematous, mononematous, erect, cylindrical, short, straight or slightly flexuous, occasionally branched, 0–3-septate, hyaline to pale brown, thick-walled. ***Conidiogenous cells*** 12.5–14 × 3.7–4.5 μm (*x̄* = 13 × 4 μm, n = 25), holoblastic, monoblastic or polyblastic, integrated, terminal, cylindrical, truncate at apex after conidial secession, hyaline to pale brown, smooth-walled. ***Conidia*** solitary, acrogenous, helicoid, rounded at apex, 80–104 μm diameter and conidial filament 6.5–8 μm wide (*x̄* = 96.5 × 7 μm, n = 20), 431–485 μm long (*x̄* = 452 μm, n = 20), loosely coiled up to 1^1^/_2_ times, becoming loosely coiled in water, septate, slightly constricted at septa, guttulate, hyaline to pale brown, smooth-walled.

##### Culture characteristics.

Conidia germinated on PDA and produced germ tubes within 12 h. Colonies on PDA reached 29 mm in diameter after 41 days of incubation at 25 °C with an irregular shape, raised surface and undulate margin, pale brown to reddish-brown; the reverse was brown to reddish-brown.

##### Material examined.

China • Hainan Province, Lingshui Lizu Autonomous County, Diaoluoshan National Nature Reserve, 18°43'N, 109°43'E, on rotting wood in a terrestrial habitat, 24 August 2021, Jian Ma, DL24 (HKAS 128911, holotype), ex-type living culture GZCC 22-2142; • *Ibid*., DL26 (GZAAS 22-2142, paratype), living culture GZCC 25-0643.

##### Notes.

In the phylogenetic analyses (Fig. 1), *Tubeufia diaoluoshanensis* formed a sister clade to *T. fangchengensis* with 55% ML statistical support. However, a comparison of nucleotides in the ITS, LSU, *tef*1-α and *rpb*2 sequence between *T. diaoluoshanensis* (GZCC 22-2142) and *T. fangchengensis* (MFLUCC 17-0047) revealed nucleotide differences of 55/931 bp (5.9%, including 24 gaps), 7/831 bp (0.8%, including one gap), 28/912 bp (3.1%, without gap) and 62/863 bp (7.2%, without gap), respectively. Moreover, *T. diaoluoshanensis* (HKAS 128911) differs from *T. fangchengensis* (HKAS 97429) by its shorter conidiophores (24–57 μm vs. up to 135 μm) and longer conidia (431–485 μm vs. 165–220 μm) (Lu et al. 2018b). Therefore, based on morphological comparisons and multi-gene phylogenetic analyses, *Tubeufia diaoluoshanensis* (GZCC 22-2142 and GZCC 25-0643) is introduced here as a new species.

## ﻿Discussion

Currently, the genus *Helicotubeufia* comprises five recognised species, including the newly-described *H. qixianlingensis*. Notably, the conidiophores and conidiogenous cells of *H. qixianlingensis* exhibit remarkable morphological similarities to those of *Tubeufia* species (Lu et al. 2018b; Ma et al. 2024). This observation underscores that, despite the critical role of molecular phylogenetic analyses, conidial morphology remains an essential feature for species delimitation within helicosporous hyphomycetes (Liu et al. 2018; Lu et al. 2018b; Ma et al. 2024). Consequently, the precise identification of these fungi necessitates a holistic approach that integrates comprehensive multi-gene phylogenetic analyses with detailed morphological assessments.

Based on molecular phylogenetic analyses and detailed morphological comparisons, Ma et al. (2024) transferred *Xenosporium* to the genus *Tubeufia*. Currently, *Tubeufia* comprises 57 recognised helicosporous species, making it the second-largest genus in Tubeufiaceae after *Helicoma*, which includes 68 species (Lu et al. 2018b; Ma et al. 2024, 2025). Taxonomic and ecological studies indicate that *Tubeufia* species predominantly occur in tropical and subtropical regions, with most records from Thailand and Hainan Province, China (Penzig and Saccardo 1897; Barr 1979; Rossman 1987; Ho et al. 2002; Tsui and Berbee 2006; Tsui 2007; Zhao et al. 2007; Boonmee et al. 2011, 2014; Chaiwan et al. 2017; Dai et al. 2017; Doilom et al. 2017; Lu et al. 2017, 2018a, 2018b, 2022, 2023; Lu and Kang 2020; Tian et al. 2022; Ma et al. 2023, 2024; 2025; Lu et al. 2025). Several species have also been reported from other southern Chinese regions, including Guangxi, Guizhou, Yunnan and Taiwan, reflecting a relatively broad, but climate-dependent distribution (Lu et al. 2017, 2018a, 2018b, 2022, 2023; Lu and Kang 2020; Tian et al. 2022; Ma et al. 2023, 2024; 2025; Lu et al. 2025). The frequent discovery of new species from similar habitats further implies that *Tubeufia* may have undergone adaptive radiation in response to microhabitat heterogeneity, particularly within decaying wood and leaf litter environments. Hence, understanding the biogeographical and ecological correlates of *Tubeufia* diversity will be essential for elucidating the evolutionary processes driving speciation within Tubeufiaceae. Integrating ecological niche modelling with molecular phylogenetics in future research may provide deeper insights into how environmental factors and substrate preferences shape the diversification of this genus.

Several helicosporous species were isolated from the same locality during concurrent field collections. Although multi-gene phylogenetic analyses confirmed that these isolates represent distinct species, they exhibit close evolutionary relationships, suggesting recent divergence or shared ecological adaptation. For instance, *Tubeufia acropleurogena* (CGMCC 3.25582) and *T. baomeilingensis* (CGMCC 3.25580) were both obtained from decaying wood in a terrestrial habitat at Baomeiling, Hainan Province, on 15 August 2021, highlighting the co-occurrence of closely-related species within the single microhabitat (Ma et al. 2024). Such sympatric occurrence of phylogenetically allied taxa implies that ecological factors, such as substrate type, microclimatic conditions and resource partitioning, may play crucial roles in maintaining species boundaries and promoting niche differentiation. Therefore, the co-occurrence of these species not only highlights the high microhabitat diversity within subtropical forest ecosystems, but also underscores the ecological complexity underlying species diversification in helicosporous hyphomycetes. Further integrative studies combining fine-scale ecological data, population-level molecular analyses and physiological assays would be valuable for elucidating the adaptive mechanisms shaping their co-existence and evolutionary trajectories.

## Supplementary Material

XML Treatment for Helicotubeufia qixianlingensis

XML Treatment for Tubeufia diaoluoshanensis

## ﻿Additional information

### Use of AI

No use of AI was reported.

### Author ORCIDs

Ting-Hong Tan https://orcid.org/0000-0002-2933-2696

Fan Gao https://orcid.org/0009-0003-9887-9729

Song Bai https://orcid.org/0000-0002-1972-2834

Chun-Fang Wu https://orcid.org/0009-0002-5166-0060

Ning-Ning Zhao https://orcid.org/0009-0004-4407-755X

Na Qiu https://orcid.org/0009-0002-1637-4720

Min Zhou https://orcid.org/0009-0009-6871-8928

Jian Ma https://orcid.org/0009-0008-1291-640X

### Data availability

All of the data that support the findings of this study are available in the main text.
